# First record of leopard-spotted goby *Thorogobiusephippiatus* (Gobiiformes, Gobiidae) above the Arctic circle

**DOI:** 10.3897/BDJ.12.e127963

**Published:** 2024-07-26

**Authors:** Vsevolod Rudyi, Cesc Gordó-Vilaseca

**Affiliations:** 1 Nord University, Bodø, Norway Nord University Bodø Norway

## Brief communication

The leopard-spotted goby *Thorogobiusephippiatus* (Lowe, 1839) distribution extends from the Western edge of the Mediterranean to the Eastern Atlantic, from Madeira to the North Sea (Costello 1992, Gerovasileiou et al. 2017, Stern et al. 2018). This species inhabits crevices and caves, and they tend to hide and withdraw at the minimum disturbance by divers or light (Holm and Mattson 1981), making its detection particularly challenging and typically restricted to observations by scuba divers (Costello 1992).

**Figure 1.**
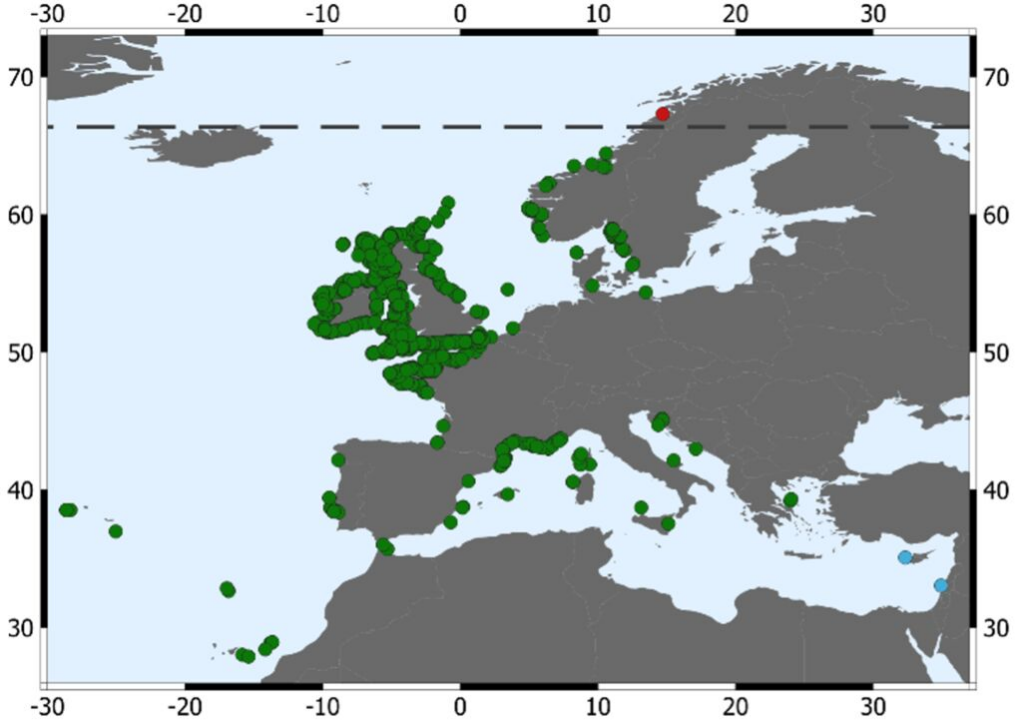
Global records of *Thorogobiusephippiatus* (GBIF.org 2024). Green dots correspond to records in GBIF. Blue dots are the observations, not in GBIF, from (Gerovasileiou et al. 2017) and (Stern et al. 2018). The red dot represents our observation, the first one above the Arctic circle (black dashed line).

**Figure 2.**
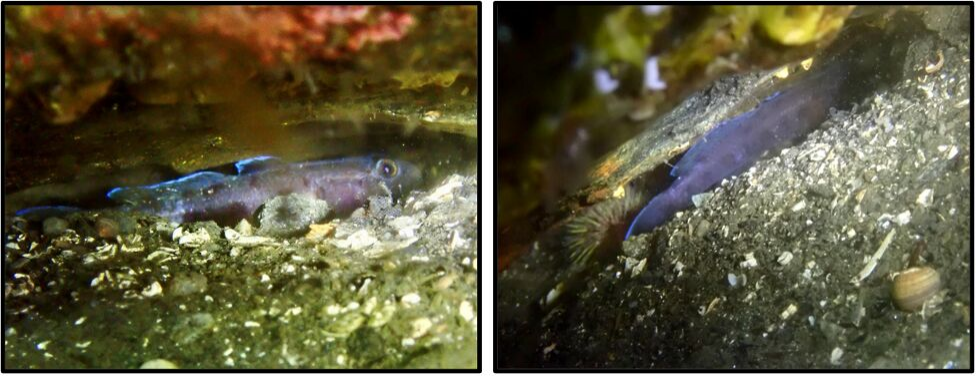
Pictures taken during the dive by CGV. The individual was photographed in a crevice at ~5 m depth on the 9^th^ and 10^th^ of April 2024.

In the last decades, new records of this species have been reported in Sweden and Norway, but never North of 64°22'24'' (Kovačić and Svensen 2018, Pethon 2005) (Fig. 1). These new records at northern latitudes could either be the result of previous lack of knowledge of this species range limit, or the result of climate warming induced northward expansion of its range, as has been observed for several other species, including the Atlantic Mackerel (*Scomberscombrus*), a pelagic species, or the spotted dragonet (*Callionymusmaculatus*), a demersal one (Gordó-Vilaseca et al. 2023). Here we document the first confirmed record of this species above the Arctic circle, with images from an individual observed during a recreational SCUBA dive in the eastern side of Saltfjorden, at 5 m depth. The coordinates of the place, where the fish was discovered, are 67°18'19.3464" N, 14°43'5.52" E (Fig. 2). The fish was observed in the upper subtidal zone, on a mixed sand and shell rubble bottom, hiding under a large overhanging rock. This micro-habitat is typical for this cryptobenthic species (Costello 1992). This find proves a wider and more northern extent than previously considered, and places the leopard-spotted goby as the seventh species of goby living above the Arctic circle (Kovačić and Svensen 2019).

**Figure 3.**
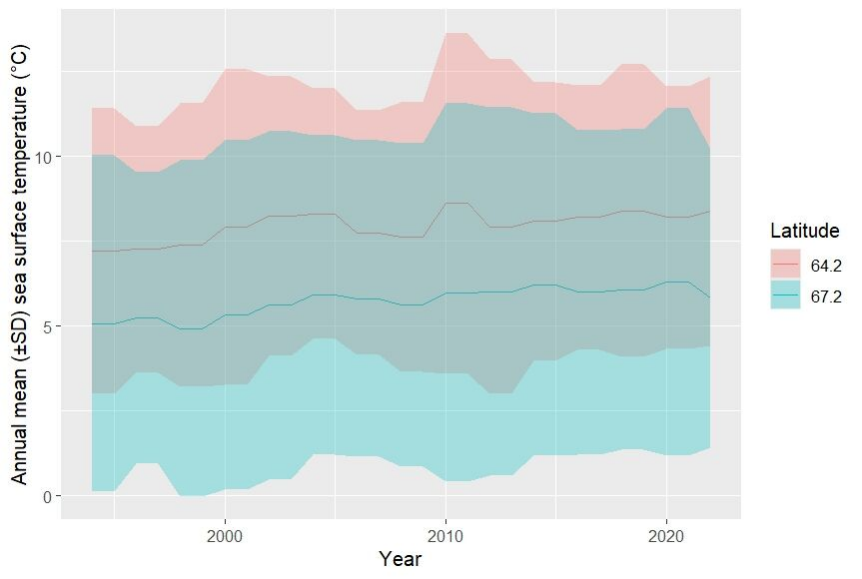
Annual mean sea surface temperature (SST) between 1994 and 2022, at the latitude of the previous northernmost record for the species, and at the latitude of this record.

The morphological and coloration evidences should be provided if the record and identification are based on morphology and coloration following the recommended best practice for reporting new fish species records (Bello et al. 2014). We document this record with pictures, and without collecting specimens, because this species’ coloration and general shape are sufficient for species identification from photographs of live specimens (Kovačić et al. 2022). Diagnosis by coloration of alive individuals: Head and body of alive specimens in water whitish, pale fawn to sandy or bluish grey (which is the case for this particular individual). Black, brown orange to brown purple blotches and spots on head and body, the largest along lateral midline and along dorsal fin bases, smaller ones on predorsal area and on head. We have accurately checked available regional fish diversity catalogues (Mecklenburg et al. 2018) and global and regional databases (GBIF.org 2024, Norwegian Biodiversity Information Centre and (2024) 2024 and OBIS 2024). The record has been documented in Artsdatabanken which in turn makes the data available in GBIF (https://www.artsobservasjoner.no/Sighting/34568198). We have regularly dived in the area over the past three years and have consulted with other scuba divers who photograph and document species in the area, and nobody had observed it before. This observation, roughly 450 km away from the previously northernmost record (Kovačić and Svensen 2018), suggests a recent range extension for the species, as has been happening for other marine fishes (Gordó-Vilaseca et al. 2023), and following the positive temperature trend of the last decades in the area (Fig. 3). Further records in regularly dived localities may confirm the spread of the species.
